# Multimodal Management of a Large Postsurgical Wound Secondary to Nodulectomy in a Dog: Application of the TIME Framework, Tie‐Over Fixation, Fluorescence Biomodulation, and Electrobioregulation

**DOI:** 10.1155/crve/6722904

**Published:** 2026-09-07

**Authors:** Sergio Eduardo Rodríguez-Aguilar, Juan Ocampo-López, Rodrigo Salomón Hernández-Aco, Diana Patricia Carreón-Camacho, María Guadalupe Ramírez-Muñoz, Armando Zepeda-Bastida

**Affiliations:** ^1^ Academic Area of Veterinary Medicine and Animal Science, Institute of Agricultural Sciences, Autonomous University of the State of Hidalgo, Tulancingo de Bravo, Hidalgo, Mexico

**Keywords:** advanced wound management, dog, electrobioregulation, fluorescence biomodulation, second-intention healing, tie-over technique, TIME framework, wound healing

## Abstract

Extensive postsurgical wounds remain a significant therapeutic challenge in small animal veterinary practice, particularly when reconstructive procedures fail and tissue viability is compromised. This report describes the successful management of a large postsurgical wound in an 8‐year‐old spayed mixed‐breed female dog following wide‐margin nodulectomy of a dorsal thoracic mass initially diagnosed cytologically as a suspected liposarcoma. Although primary closure using an H‐plasty reconstructive technique was initially performed, progressive central necrosis developed at the surgical margins during the postoperative period. On postoperative Day 3 (POD 3), the wound exhibited tissue necrosis and serosanguineous exudation, necessitating management by second‐intention healing. Following surgical debridement, the wound measured approximately 143 cm^2^ and was subsequently managed using a multimodal advanced wound care protocol based on the principles of the TIME framework, combined with tie‐over dressing fixation, advanced wound dressings, fluorescence biomodulation, electrobioregulation, and topical regenerative therapies. Histopathological examination of the excised tissue ultimately revealed chronic‐active pyogranulomatous panniculitis with abundant fibrosis, ruling out the initial suspicion of neoplasia. Progressive wound contraction, granulation tissue formation, and epithelialization were observed throughout the treatment period, with serial measurements demonstrating a reduction from 143 cm^2^ to complete closure over approximately 16 weeks. No evidence of local infection, recurrent tissue necrosis, wound dehiscence, or systemic complications was detected during follow‐up. Complete wound healing was achieved with satisfactory functional and cosmetic outcomes, without the need for additional reconstructive surgery. This case highlights the potential clinical value of a multimodal, individualized approach to managing extensive, complicated wounds in dogs. It suggests that sequentially integrating the TIME framework, tie‐over fixation, fluorescence biomodulation, and electrobioregulation may offer a useful therapeutic alternative when conventional reconstructive techniques are unsuccessful.

## 1. Introduction

Extensive and complex wounds continue to pose a significant therapeutic challenge in small animal veterinary medicine, particularly when postsurgical complications prevent primary closure or compromise tissue viability. Extensive tissue loss, excessive tension at wound edges, dead space formation, and secondary necrosis can delay healing, increase the risk of infection, and adversely affect patient well‐being. In these cases, implementing advanced wound management strategies is essential to promote tissue repair and optimize clinical prognosis [1–5]. Healing by secondary intention is a therapeutic alternative when conventional reconstructive techniques fail to restore tissue continuity adequately. However, this process is often prolonged and requires continuous assessment of the wound bed, as well as interventions to control inflammation, reduce bacterial contamination, promote granulation tissue formation, and stimulate epithelialization. In this context, the TIME model (Tissue management, Infection/Inflammation control, Moisture balance, and Edge advancement) has become a widely used tool for the systematic preparation of the wound bed and clinical decision‐making during the healing process [5, 6].

Recent advances in wound management have considerably expanded the therapeutic options available for treating complex wounds. These include advanced wound dressings, hemostatic sponges, reactive tissue dressings (RTDs), paraffin‐impregnated gauzes, topical regenerative agents, the tie‐over dressing fixation technique, fluorescence biomodulation using fluorescent energy, and electrobioregulation, all of which have been associated with improved wound healing outcomes in both human and veterinary medicine, strategies that have been shown to promote angiogenesis, cell proliferation, collagen synthesis, and granulation tissue formation, contributing to improved clinical outcomes in extensive or difficult‐to‐treat lesions [7–16]. However, the available clinical evidence on the sequential and integrated application of these therapeutic modalities in veterinary patients remains limited. Therefore, this report describes the multimodal management and clinical evolution of an extensive postsurgical wound in a dog using a sequential therapeutic approach based on the TIME framework, tie‐over dressing fixation, fluorescence biomodulation, and electrobioregulation.

## 2. Case Presentation

**Figure 1:**
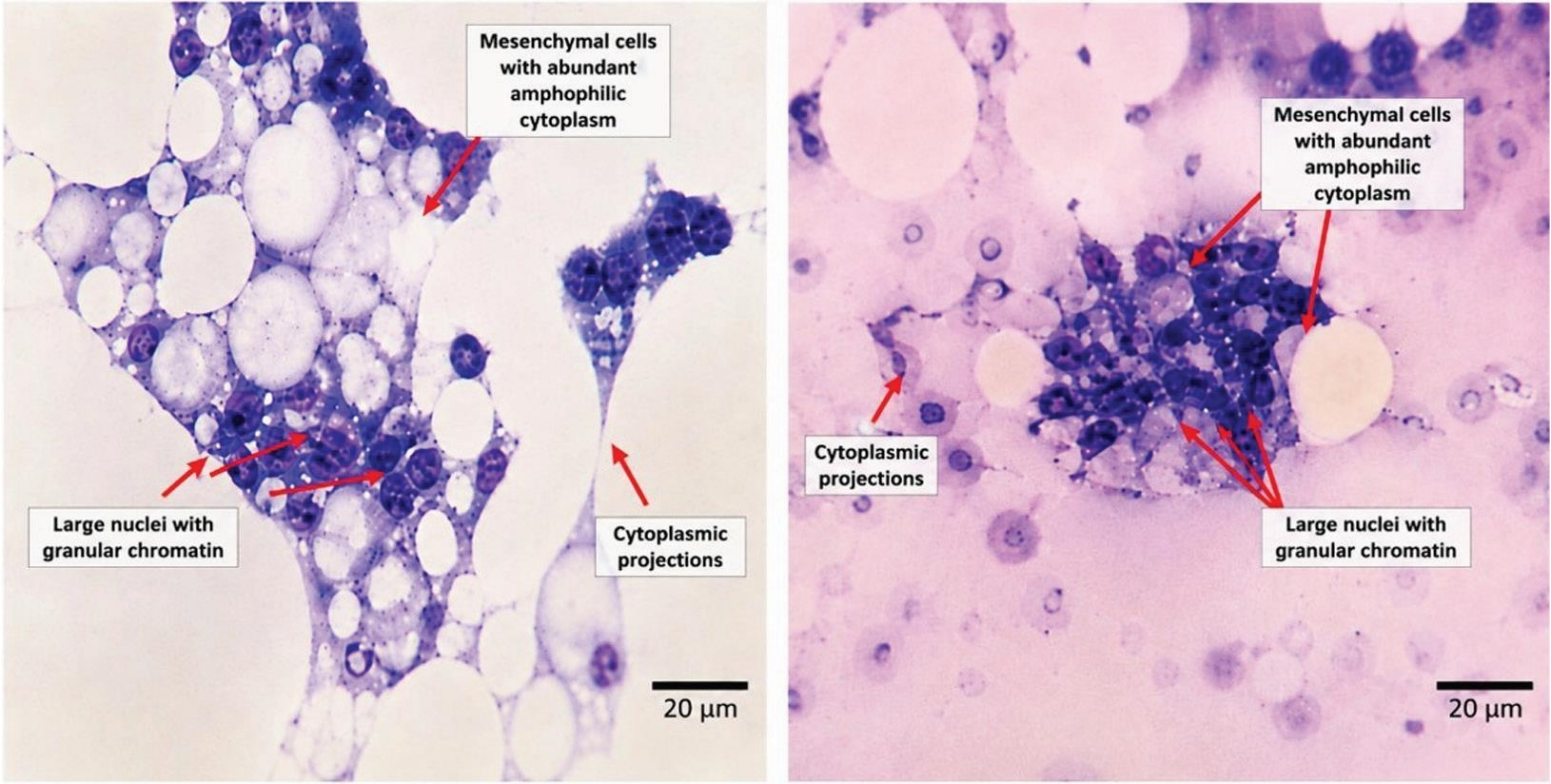
Cytological evaluation of the mass by fine‐needle aspiration (FNA). Mesenchymal cells with abundant amphophilic cytoplasm, cytoplasmic projections, and large nuclei with granular chromatin are observed, findings consistent with a presumptive diagnosis of mesenchymal neoplasm.

An 8‐year‐old, spayed, mixed‐breed female dog (*Canis lupus familiaris*) was presented for evaluation of a mass located in the dorsal thoracic region. The mass measured approximately 6 cm in diameter, firm, with irregular borders, and was nontender to palpation. According to the owner, the mass had been present for approximately 6 weeks. Fine‐needle aspiration (FNA) cytology revealed a moderately cellular population composed of mesenchymal cells with cytoplasmic projections, abundant amphophilic cytoplasm, and large nuclei with granular chromatin and prominent nucleoli. These cytological findings were considered highly suggestive of a mesenchymal neoplasm, supporting a presumptive diagnosis of liposarcoma and guiding subsequent surgical management. (Figure 1).

**Figure 2:**
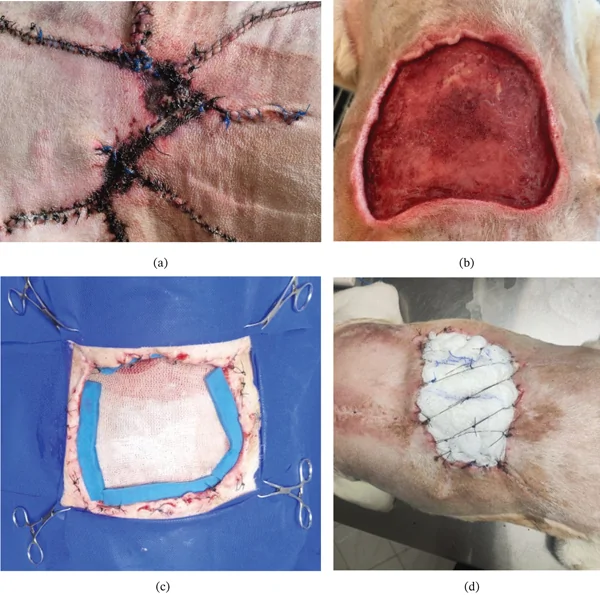
Initial wound assessment and management following nodulectomy. (A) On postoperative Day 3 (POD 3), necrosis of the surgical margins with serosanguineous exudate was observed, prompting a change in the therapeutic strategy toward second‐intention healing. (B) On POD 5, following surgical debridement of devitalized tissue, the wound measured approximately 13 × 11 cm (143 cm^2^). It exhibited viable margins and a prepared wound bed. (C) Application of a reactive tissue dressing (RTD) and hemostatic foam sponge (Surgispon) for wound bed management and exudate control. (D) Fixation of the dressings using the tie‐over technique to maintain close contact with the wound bed, minimize movement, and control dead space.

Based on clinical and cytological findings, surgical excision of the mass was performed via nodulectomy with wide margins. Due to the limited skin laxity in the affected area, primary closure was initially attempted using an H‐plasty reconstructive technique. However, during the procedure, the size of the surgical defect prevented tension‐free closure. Therefore, it was necessary to modify the intraoperative reconstructive approach and submit the sample for histopathological evaluation. On postoperative Day 3 (POD 3), the patient was re‐evaluated, revealing necrosis in the central portion of the surgical margins accompanied by serosanguineous exudate. Due to compromised tissue viability and lesion characteristics, a new primary closure was ruled out, and an advanced wound management protocol using secondary intention healing was implemented (Figure 2A).

During the first advanced wound management (POD 5), a systematic assessment was performed following the principles of the TIME model. Devitalized tissue was surgically debrided until viable wound margins were obtained. This procedure resulted in a clean and well‐prepared wound bed suitable for advanced wound management. The lesion presented an oval morphology with approximate dimensions of 13 × 11 cm, corresponding to an estimated surface area of 143 cm^2^ (Figure 2B).

Following wound cleansing with 0.01% chlorhexidine diluted in sterile physiological saline, an RTD and a hemostatic foam sponge were applied. The materials were secured using the tie‐over technique with 0‐gauge nylon sutures to promote close contact between the dressing and the wound bed, minimize movement, and control dead space (Figure 2C,D). Additionally, a copper‐impregnated gown was applied to protect the wound and reduce the risk of external contamination.

**Figure 3:**
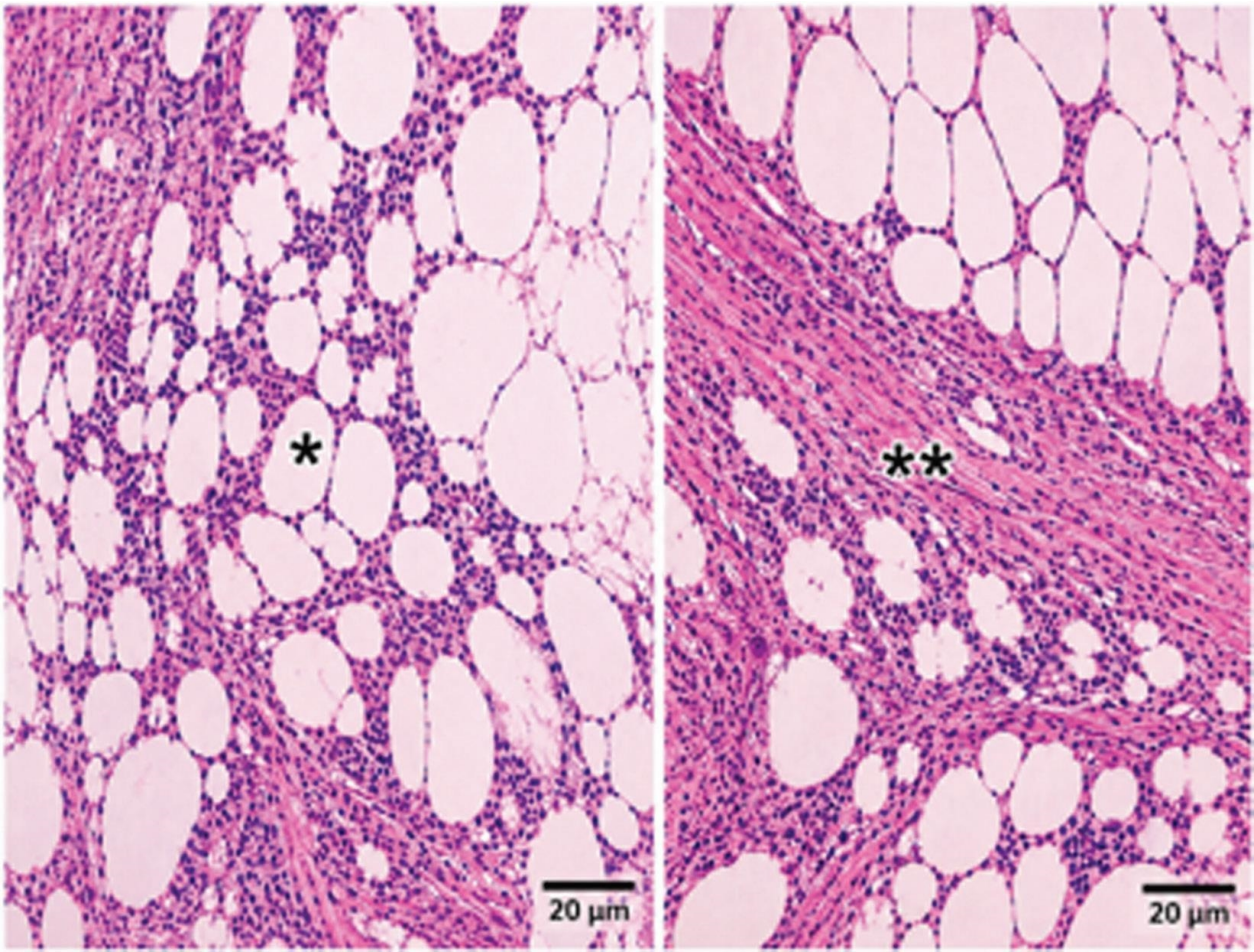
Histopathological findings of the mass removed by excisional biopsy. A pyogranulomatous inflammatory infiltrate (∗), accompanied by abundant fibrosis (∗∗), is observed consistently with focal, moderate, active chronic pyogranulomatous panniculitis.

On POD 12, the histopathological results were received. Histopathological examination revealed abundant inflammatory infiltrates composed of degenerated neutrophils, foamy macrophages, lymphocytes, and reactive fibroblasts, in addition to neovascularization and extensive fibrosis. The final diagnosis was focal moderate active chronic pyogranulomatous panniculitis with extensive fibrosis, ruling out the initial presumptive diagnosis of liposarcoma (Figure 3). During this period, the lesion showed favorable evolution, reducing its dimensions to approximately 11 × 9 cm.

**Figure 4:**
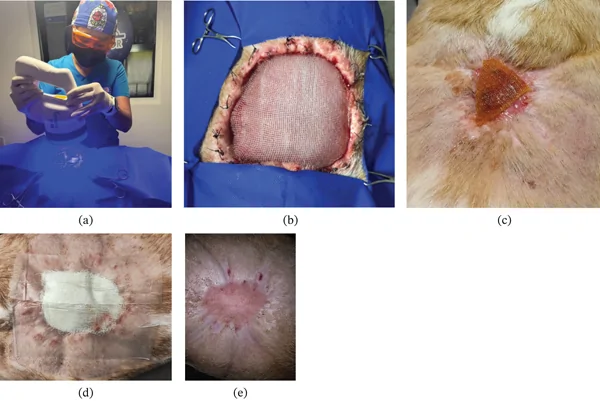
Clinical evolution of the wound during multimodal management and second‐intention healing. (A) Application of fluorescence biomodulation (Phovia) during the proliferative phase of wound healing. (B) Evolution of the wound during the granulation phase, with application of *Triticum vulgare* extract (Italdermol) and paraffin‐impregnated gauze (Jelonet) as part of the sequential wound management protocol. (C) On POD 61, the wound measured approximately 4.5 × 3 cm (13.5 cm^2^), representing a reduction of more than 90% from the initial wound area; topical phytostimulin and a breathable dressing were subsequently used. (D) By approximately POD 90, the wound measured approximately 2 × 1 cm (2 cm^2^), with marked epithelialization and tissue remodeling; a medical‐grade honey dressing was incorporated during this stage. (E) Between POD 110 and POD 120, the wound closed completely, with adequate epithelialization and scar remodeling and no evidence of local complications.

Between POD 15 and POD 30, the initial advanced wound management protocol was maintained due to persistent exudate and dead space, although both gradually decreased. After confirming the nonneoplastic nature of the lesion, fluorescence biomodulation (Phovia) was incorporated, applied after cleansing and before dressing application (Figure 4A).

Between POD 31 and POD 60, a significant reduction in the lesion was observed, with dimensions of approximately 8 × 6 cm and an estimated surface area of 48 cm^2^. The reduction in exudate allowed modification of the treatment protocol. Electrobioregulation was incorporated followed by fluorescence biomodulation and the sequential application of topical agents designed to promote enzymatic debridement and tissue regeneration. Subsequently, impregnated gauze was applied, and a partial wound closure was performed again using the tie‐over technique (Figure 4B).

On POD 61, the lesion measured approximately 4.5 × 3 cm, corresponding to a surface area of 13.5 cm^2^, representing more than a 90% reduction from the initial area. Clinically, adequate scar tissue formation was observed, with no pain or associated systemic signs. Due to the favorable evolution, the anchoring sutures were removed, and a protocol based on topical phytostimulin and fixation with a breathable dressing was implemented (Figure 4C).

By approximately POD 90, the wound reached approximate dimensions of 2 × 1 cm (2 cm^2^), with marked epithelialization and tissue remodeling. At this stage, a medical‐grade honey dressing was added to complement the healing protocol while maintaining local protection with secondary dressings (Figure 4D).

Finally, between POD 110 and POD 120, complete wound healing of the lesion was observed, with adequate epithelialization, remodeling of the scar tissue, and absence of exudate or local complications. The patient experienced a satisfactory functional and esthetic recovery and was therefore discharged with an excellent long‐term prognosis (Figure 4E).

**Figure 5:**
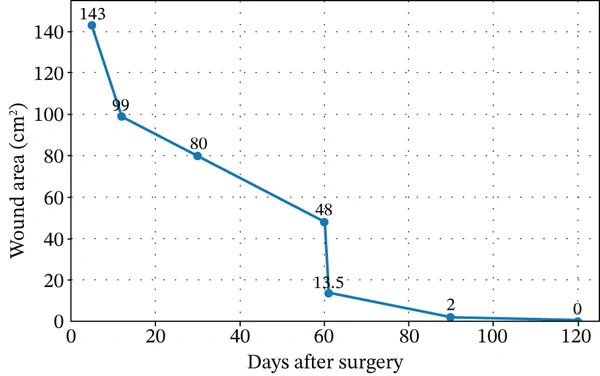
Progressive reduction of wound area during multimodal wound management. The wound measured approximately 143 cm^2^ on POD 5 after surgical debridement and subsequently decreased to 99 cm^2^ (POD 12), 80 cm^2^ (POD 30), 48 cm^2^ (POD 60), 13.5 cm^2^ (POD 61), and 2 cm^2^ (POD 90). Complete wound closure was achieved between POD 110 and 120 following the implementation of a multimodal treatment protocol based on the TIME framework, tie‐over fixation, fluorescence biomodulation, and electrobioregulation. Values correspond to clinical measurements obtained during follow‐up evaluations.

Overall, serial wound measurements documented progressive contraction throughout the follow‐up period. Following surgical debridement on POD 5, the wound measured approximately 143 cm^2^, decreasing to 99 cm^2^ on POD 12, 80 cm^2^ on POD 30, 48 cm^2^ on POD 60, 13.5 cm^2^ on POD 61, and 2 cm^2^ by approximately POD 90 (Figure 5). Complete epithelialization and wound closure were achieved between POD 110 and POD 120. Throughout the follow‐up period, no clinical evidence of wound infection, recurrent tissue necrosis, wound dehiscence, or systemic complications was observed. The final functional and cosmetic outcomes were considered satisfactory.

## 3. Discussion

The management of extensive postsurgical wounds remains a significant challenge in small animal surgery, particularly when reconstructive procedures fail or when vascular compromise leads to tissue necrosis. In the present case, H‐plasty was initially selected as a reconstructive option to facilitate primary closure of the dorsal thoracic defect by redistributing tension through advancement of adjacent skin. However, the extent of the surgical defect and limited local skin laxity prevented adequate tension‐free closure, increasing tension at the wound margins and potentially compromising local perfusion. The subsequent development of central necrosis at the surgical margins indicated that the reconstructive approach was not suitable for the characteristics of this defect. Similar complications have been described in wounds subjected to excessive tension or inadequate vascular supply, where tissue ischemia may compromise healing and necessitate alternative approaches such as second‐intention wound management [3, 6, 15, 16].

A key factor in the favorable evolution of this case was the systematic application of the TIME framework, which facilitated sequential management of nonviable tissue, inflammation, moisture balance, and wound edge progression. Wound bed preparation has been widely recognized as a cornerstone of advanced wound management because it creates an optimal microenvironment for granulation tissue formation and epithelial migration. In the present case, repeated debridement, exudate control, and maintenance of an adequate moisture balance were associated with progressive wound contraction and the development of healthy granulation tissue, supporting previous reports that emphasize the clinical utility of the TIME approach in complex wounds [5, 6]. The favorable evolution observed in this patient was also associated with the sequential use of advanced wound dressings specifically selected according to the wound healing stage. Moisture‐retentive dressings, foam materials, and RTD have been shown to improve exudate control, promote autolytic debridement, and facilitate granulation tissue formation, thereby optimizing the wound microenvironment for healing [1, 3, 12, 15].

The tie‐over technique also played an important role throughout the treatment period. Extensive dorsal wounds are particularly difficult to manage because of constant movement, dead‐space formation, and challenges associated with maintaining dressings in close contact with the wound bed. By providing uniform pressure and stable fixation of the wound dressings, the tie‐over system contributed to exudate control and protection of the wound surface. Similar benefits have been reported in reconstructive and wound management procedures in dogs and cats, where tie‐over fixation improves dressing stability and promotes the development of granulation tissue [15, 16]. During the initial stages of treatment, the use of a hemostatic foam sponge contributed to exudate management and local wound stabilization. These materials act as temporary matrices that facilitate fluid absorption and support clot formation, which improves the local wound environment required for tissue repair [7].

Chronic and extensive wounds are particularly susceptible to bacterial biofilm formation, which may delay healing by maintaining persistent inflammation and protecting microorganisms from host immune responses and topical antimicrobial agents. Effective wound bed preparation through repeated debridement, moisture balance, and the appropriate selection of advanced wound dressings are considered essential strategies for disrupting biofilms and promoting tissue repair. Although microbiological biofilm analysis was not performed in the present case, the sequential implementation of the TIME framework and advanced wound care principles may have contributed to minimizing bacterial burden and creating a favorable environment for progressive healing [17, 18].

Another notable aspect of this case was the incorporation of fluorescence biomodulation during the proliferative phase of healing. Recent veterinary studies have demonstrated that fluorescence biomodulation can stimulate fibroblast proliferation, collagen synthesis, angiogenesis, and modulation of inflammatory mediators, thereby improving tissue repair and reducing healing times. Experimental and clinical evidence in dogs suggests that fluorescence biomodulation may accelerate wound healing and decrease postoperative inflammation and exudate production, although standardized treatment protocols remain lacking. In the present case, fluorescence biomodulation was introduced after histopathological confirmation of a nonneoplastic lesion and coincided with progressive wound contraction and improved tissue quality, supporting its potential role as an adjunctive therapy in complex wound management [13, 14, 19–22]. In addition to fluorescence biomodulation, several topical regenerative agents were incorporated throughout the healing process. Products containing *Triticum vulgare* derivatives have been associated with stimulation of fibroblast activity, collagen synthesis, and epithelial regeneration, whereas enzymatic debridement agents such as collagenase facilitate selective removal of devitalized tissue while preserving viable structures. These therapies may have supported the progressive wound contraction and tissue remodeling observed in the present case [8–10].

Electrobioregulation was incorporated during the later stages of healing as part of the multimodal treatment strategy. Endogenous bioelectric signals are known to influence cellular migration, angiogenesis, collagen deposition, and tissue remodeling. Previous studies have shown that electrical stimulation can enhance wound repair through activation of signaling pathways involved in cell proliferation and tissue regeneration. Although the individual contribution of electrobioregulation cannot be determined in a single‐case report, its use coincided with accelerated wound contraction and progressive epithelialization, suggesting that modulation of bioelectrical activity may represent a useful complementary approach in selected veterinary patients [11].

During the final stages of healing, phytostimulin‐based formulations, enzymatic debridement agents, and protective dressings were incorporated to support epithelial maturation and scar remodeling. Collagenase‐based products facilitate selective removal of devitalized tissue while preserving viable structures and improving wound bed preparation. Likewise, phytostimulin formulations derived from *T. vulgare* have been associated with stimulation of fibroblast proliferation, extracellular matrix production, and epithelial regeneration. Although evidence regarding these products in veterinary medicine remains limited, their use is common in clinical practice as adjunctive therapies for wound management and tissue repair and may have contributed to the favorable healing outcome observed in this patient [8–10].

An important finding of this report was the successful management of a wound measuring approximately 143 cm^2^ without the need for additional reconstructive surgery. Large dorsal defects are frequently associated with prolonged healing periods and may require skin flaps, axial pattern flaps, or skin grafting procedures. In contrast, the present patient achieved complete healing through second‐intention wound management combined with advanced wound care techniques. The wound area decreased from 143 cm^2^ to complete closure within approximately 16 weeks, representing a progressive reduction of 100% of the original wound surface. These findings compare favorably with previous reports describing conservative management of extensive wounds managed conservatively and highlight the potential benefits of combining multiple evidence‐based therapeutic modalities [4, 5, 12, 15, 16].

Unlike previously published veterinary reports, which have primarily described the isolated application of fluorescence biomodulation, advanced wound dressings, or reconstructive techniques, the present case documents the successful sequential integration of the TIME framework, tie‐over fixation, fluorescence biomodulation, electrobioregulation, and topical regenerative therapy following failure of an H‐plasty reconstructive procedure. To the authors′ knowledge, few reports have described the management of a postsurgical wound of approximately 143 cm^2^ using this multimodal therapeutic strategy until complete healing was achieved by second intention without additional reconstructive surgery [13, 14, 19–22]. This comprehensive and individualized approach may represent a practical therapeutic alternative for the management of extensive and complicated postsurgical wounds in dogs. Future prospective studies are warranted to determine whether this multimodal strategy provides superior outcomes compared with conventional wound management protocols.

Several limitations should be acknowledged. As a single‐case report, it is not possible to determine the relative contribution of each therapeutic intervention to the outcome. The sequential use of advanced dressings, tie‐over fixation, fluorescence biomodulation, electrobioregulation, and topical regenerative agents precludes identification of which modality had the greatest influence on healing. Furthermore, we did not perform standardized objective assessments of tissue perfusion, collagen deposition, or inflammatory biomarkers. Therefore, controlled clinical studies are required to evaluate the efficacy of these therapies individually and in combination.

## 4. Conclusions

This case documents the successful second‐intention healing of an extensive postsurgical wound following failure of an initial reconstructive procedure and subsequent surgical margin necrosis. The favorable clinical outcome suggests that a structured, individualized, multimodal wound management approach may be a useful therapeutic option for extensive, complicated wounds in dogs when conventional reconstructive techniques are unsuccessful. However, because this single‐case report involves multiple sequential interventions, further controlled studies are needed to evaluate the efficacy and reproducibility of this therapeutic approach.

## Author Contributions

S.E.R‐A.: data curation, formal analysis, investigation, supervision. J.O‐L.: investigation, formal analysis, supervision. R.S.H‐A.: formal analysis, supervision. D.P.C‐C.: data curation, resources. M.G.R‐M.: formal analysis. A.Z‐B.: conceptualization, data curation, investigation, writing—review and editing.

## Funding

No funding was received for this manuscript.

## Consent

Written informed consent was obtained from the patient′s owner for publication of the clinical information and accompanying images.

## Conflicts of Interest

The authors declare no conflicts of interest.

## Data Availability

Research data are not shared.
